# An Enhanced Differential Evolution Algorithm with Bernstein Operator and Refracted Oppositional-Mutual Learning Strategy

**DOI:** 10.3390/e24091205

**Published:** 2022-08-29

**Authors:** Fengbin Wu, Junxing Zhang, Shaobo Li, Dongchao Lv, Menghan Li

**Affiliations:** 1 State Key Laboratory of Public Big Data, College of Computer Science and Technology, Guizhou University, Guiyang 550025, China; 2 State Key Laboratory of Public Big Data, Guizhou University, Guiyang 550025, China; 3 School of Mechanical Engineering, Guizhou University, Guiyang 550025, China

**Keywords:** refracted oppositional learning, mutual learning, refracted oppositional-mutual learning, differential evolution, Bernstein operator, CEC 2019 and 2020

## Abstract

Numerical optimization has been a popular research topic within various engineering applications, where differential evolution (DE) is one of the most extensively applied methods. However, it is difficult to choose appropriate control parameters and to avoid falling into local optimum and poor convergence when handling complex numerical optimization problems. To handle these problems, an improved DE (BROMLDE) with the Bernstein operator and refracted oppositional-mutual learning (ROML) is proposed, which can reduce parameter selection, converge faster, and avoid trapping in local optimum. Firstly, a new ROML strategy integrates mutual learning (ML) and refractive oppositional learning (ROL), achieving stochastic switching between ROL and ML during the population initialization and generation jumping period to balance exploration and exploitation. Meanwhile, a dynamic adjustment factor is constructed to improve the ability of the algorithm to jump out of the local optimum. Secondly, a Bernstein operator, which has no parameters setting and intrinsic parameters tuning phase, is introduced to improve convergence performance. Finally, the performance of BROMLDE is evaluated by 10 bound-constrained benchmark functions from CEC 2019 and CEC 2020, respectively. Two engineering optimization problems are utilized simultaneously. The comparative experimental results show that BROMLDE has higher global optimization capability and convergence speed on most functions and engineering problems.

## 1. Introduction

Recently, numerical optimization has become a trending research topic of interest for many researchers and is broadly used to handle many engineering optimization problems, such as mobile robots path planning [1], vehicle problem [2], and task scheduling [3]. These optimization problems can be expressed as NP-Hard problems, which are difficult to derive high-quality solutions by traditional approaches owing to the reliance of traditional methods on the choice of starting points and vulnerability to optimal local problems [4,5]. Fortunately, the meta-heuristic algorithms (MAs) have the features of high efficiency, low demands for the starting point, and robustness [6]. These overcome the limitations of traditional approaches to addressing NP problems. In the past decades, tremendous MAs have been suggested to handle numerical optimization tasks, such as differential evolution (DE) [7], PSO [8], SA [9], cockroach swarm optimization [10], and so on. Among them, DE, a population-based MA, is extensively utilized in the parameter training of neural networks [11], problem prediction [12], and path planning of unmanned aerial vehicles [13], etc., because of its features such as simple model, easy execution, stronger search capability, and robustness. Regrettably, DE is susceptible to the control parameters and has drawbacks of easily falling into local optimum as well as low convergence.

Control parameters setting may impact the convergence performance of MAs. Given this, investigators have developed various strategies for DE algorithms. For instance, the authors in [14] propose a quantization orthogonal crossover operator on the basis of an orthogonal design, where the operator crossover probabilities are considered as structural parameters. Unfortunately, its parameters are set artificially, and the stability of the algorithm is difficult to be guaranteed. To further improve algorithm performance, parameter adaptation approaches have been proposed. The authors in [15] introduce an automatic adaptation parameter mechanism that performs depending on the deviation of the objective function between the optimal individuals and the total population in the preceding generation, which helps improve the performance of the mutation stage. An adaptive parameter DE algorithm is reported in [16], which achieves parameter adaption using Q-learning. Furthermore, parametric random selection methods have also been studied by many researchers. To list a few, the authors in [17] present a self-adaptive parameters DE algorithm, where parameters (CR and F) are randomly selected from a list of stored success parameters or generated at random. Further, the authors in [18] introduce a zoning strategy to obtain the optimal combination of control parameters, and an adaptive DE algorithm having zoning evolution is proposed. Specifically, an easily controllable and non-recursive Bernstein-search DE algorithm (BSDE) is reported in [19], which is not required to control the parameter setting operation and has a flexible random mutation and crossover process. Therefore, BSDE is simpler than other parameter tuning algorithms and aids in saving the time of algorithm search. Nevertheless, the algorithm may still be locally optimal and low convergence. Thus, one motivation is raised for this paper.

Moreover, it is critical that the MAs should balance exploration and exploitation [20,21]. For this, the opposition-based learning (OBL) strategies are the powerful search framework [22,23,24]. The OBL mechanism is to explore improved candidate solutions by considering both the original points and their opposite counterparts. It is appropriate for population initialization of MAs and has performed significantly in improving the convergence of the MAs [25]. Hence, many variants of the OBL strategy have been reported to strengthen MAs in terms of trade-offs between exploration and exploitation. The authors in [26] propose an opposition-based DE having a protective jumping rate, which achieves stopping the opposite operator when the success rate of the opposite individual falls to a constant threshold. In [27], OBL with the current optimum DE algorithm is proposed, and its concept is that instead of using the center point to calculate the opposite point, the best point of the current point is utilized. Meanwhile, OBL variants based on expanded search space have also been extensively studied. To be specific, in [28], a refracted oppositional learning (ROL) strategy is incorporated into the artificial bee colony algorithm, promoting the diversity of the population and guiding it to explore the global optimal solution. Based on the ROL strategy, the authors in [29] propose a cuckoo search algorithm with refraction learning, improving the capability of cuckoo search to avoid local optimal positions. Regrettably, their suitable scale factors are difficult to select. Furthermore, a neighborhood opposition-based DE is developed in [30] by executing the Gaussian perturbation operation around the opposite point, and its search neighborhood is further expanded. In [31], a dynamic OBL mechanism with asymmetric search space is proposed, which facilitates the exploitation and exploration capabilities. However, the newly introduced weights that need to be adjusted may bring an additional burden for applications. To overcome this shortcoming, an enhanced basic DE algorithm is developed in [32] by integrating the OBL strategy and mutual learning (ML) strategy, called the oppositional-mutual learning DE (OMLDE) algorithm. Nevertheless, it may still suffer from the difficulty of choosing the suitable control parameters for DE and low convergence accuracy. Thus, another motivation is derived here.

Based on the no free lunch theorem, no single algorithm can be suitable for all optimization problems [33]. Therefore, it is valuable for modifying existing algorithms, developing new algorithms, and mixing different algorithms to obtain better results in practical applications. Inspired by the above discussions, with the help of Bernstein-search, ROL and ML strategies, an enhanced DE algorithm with both Bernstein operator and refracted oppositional-mutual learning strategy with a dynamic adjustment factor mechanism (called BROMLDE) is proposed to achieve fast convergence, jumping out of local optimum as well as reduced parameter selection in this paper. Highlights of this paper are as follows:

(1) The ROL strategy with a dynamic adjustment factor changed with function evaluation quantity is presented, which facilitates jumping out of the local extremum space.
(2) Integrating ML into ROL strategy, a novel refracted oppositional-mutual learning (ROML) strategy is proposed for better trade-off algorithm exploration and exploitation.
(3) BROMLDE can be easily operated in parallel, achieving a rapid search. Moreover, BROMLDE is a partially elitist selective method since it uses both the fittest points and global minimizer solution in its system equations.
(4) Compared with BSDE [19], BROMLDE integrates the ROML into the initialization phase of the population and the generation phase of jumping.
(5) Different from the OMLDE algorithm [32], BROMLDE does not require the adjustment of intrinsic control parameters.
(6) Several numerical experiments are investigated on the CEC 2019 and CEC 2020 benchmarks to validate the function optimization performance. Additionally, two constrained engineering problems are used to verify the feasibility of the proposed BROMLDE.

The arrangement of the rest of this paper is as below: Section 2, the differential evolution is presented. Section 3, the developed BROMLDE is stated. Numerical experiments and results analysis are described in Section 4. Lastly, conclusions and future work are provided in Section 5.

## 2. Differential Evolution Algorithm

### 2.1. The Structure of Typical DE

A typical DE [7] includes the mutation operator, crossover phase, and selection phase. The population $P_{g}$ is constructed as $P_{g}=[X_{1,g},X_{2,g},\ldots ,X_{N_{p},g}]$ for any generation (g), $P_{g}$ is derived from Equation (1), where $X_{i,g}$ is the $i^{th}$ individual vector. Each $X_{i,g}$ has D dimension, where $i=1,2,\ldots ,N_{p}$, thus $X_{i,g}={\left[x_{1i,g},x_{2i,g},\ldots ,x_{Di,g}\right]}^{T}.$

(1) $$P_{(i,j),g}\sim U\left(low_{j,g},up_{j,g}\right)|j=1,2,\ldots ,D,$$

in which $N_{p}$ is the population $P_{g}$ size, and D denotes the population dimension.

#### 2.1.1. Mutation Operator

A mutation operator can generate a mutant vector $V_{i,g}={\left[v_{1i,g},v_{2i,g},\ldots ,v_{Di,g}\right]}^{T}$. For instance, a classical mutation strategy “DE/rand/1” is given as follows:

(2) $$V_{i,g}=F\left(X_{d1,g}-X_{d2,g}\right)+X_{d3,g},i\neq d1\neq d2\neq d3,$$

where three individuals $\;X_{d1,g},X_{d2,g}$, and $X_{d3,g}$ are randomly obtained in $P_{g}$. The mutation scale factor F is usually in [0,1].

#### 2.1.2. Crossover Phase

The trail vector $U_{i,g}={\left[u_{1i,g},u_{2i,g},\ldots ,u_{Di,g}\right]}^{T}$ is constructed according to $X_{i,g}$ and $V_{i,g}$ in the process of crossover operator. The vector is updated by Equation (3):

(3) $$u_{ji,g}=\{\begin{array}{ll} v_{ji,g}, & \;if\;r_{j}⩽CR\;\|j=n_{j},\\ x_{ji,g}, & \;otherwise,\; \end{array}$$

where $r_{j}\in [0,1]$, the crossover rate CR is usually in [0,1], and the random number $n_{j}$ is chosen in [1:D].

#### 2.1.3. Selection Phase

For this phase, the individuals $X_{i,g+1}$ are generated by using Equation (4):

(4) $$X_{i,g+1}=\{\begin{array}{ll} U_{i,g}, & \;if\;f\left(U_{i,g}\right)<f\left(X_{i,G}\right),\\ X_{i,g}, & \;otherwise,\; \end{array}$$

where f(⋅) is a fitness function. The population $P_{g+1}=[X_{1,g+1},X_{2,g+1},\ldots ,X_{N_{p},g+1}]$ can be obtained.

## 3. The Proposed BROMLDE Algorithm

ROL strategy combining the refraction principle [28,34] from physics with an OBL strategy is a strong method to strengthen MAs [28,29,34,35]. In this paper, the ROL strategy is applied to augment the performance of the BROMLDE algorithm. In addition, the ROL strategy and ML strategy are combined to achieve improved exploitation capacity.

### 3.1. Bernstein Polynomials

The Bernstein polynomials can be utilized to consistently approximate a continuous function on a closed range, where polynomials of $2^{nd}$ degree [19,36] are defined using the Equations (5) and (6):

(5) $$B_{p,m}(t)=\left(\begin{array}{c} m\\ p \end{array}\right)t^{p}{\left(1-t\right)}^{m-p},p=0,1,\ldots ,m,$$

where $\left(\begin{array}{c} m\\ p \end{array}\right)=\frac{m!}{p!(m-p)!}$. The $2^{nd}$ degree Bernstein polynomials are defined as Equation (6). For p<0 and p>m, $B_{p,m}=0.$

(6) $$\left\{ \begin{array}{l} B_{0,2}\left(t\right)={\left(1-t\right)}^{2},\\ B_{1,2}\left(t\right)=2t\left(1-t\right),\\ B_{2,2}\left(t\right)=t^{2}. \end{array}\right.$$

**Figure 1:**
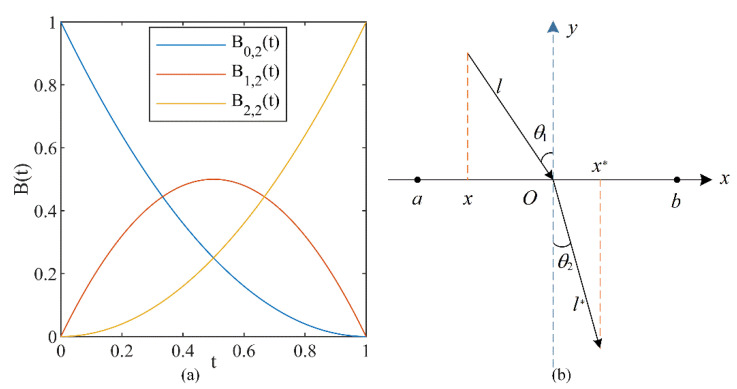
(**a**) The $2^{nd}$ degree Bernstein polynomials; (**b**) the definition of ROL strategy.

Figure 1a shows the $2^{nd}$ degree Bernstein polynomials when 0≤t≤1.

### 3.2. ROL Strategy

The principle of ROL is to calculate the fitness values for the current solution and their ROL solution and to select a superior solution by comparing the fitness values and further iterating. The ideology of ROL is shown in Figure 1b.

In Figure 1b, the x-axis is the dividing line, the normal is the y-axis, the point O is the midpoint of the search range [a,b], and the angles of incidence and refraction are $\theta_{1}$ and $\theta_{2}$, respectively, as well as l and $l^{*}$ indicating the length of incidence and refraction light, respectively. x indicates a point in the region [a,b], and $x^{*}$ stands for the reverse position of the point x. The geometric relationship of the lines in Figure 1b is expressed below:

(7) $$sin\theta_{1}=\left(\left(a+b\right)/2-x\right)/l,$$

(8) $$sin\theta_{2}=\left(x^{*}-(a+b)/2\right)/l^{*}.$$

The refraction rate η is defined by using Equation (9):

(9) $$\eta =\frac{sin\theta_{1}}{sin\theta_{2}}=\frac{l^{*}\left(\left(a+b\right)/2-x\right)}{l\left(x^{*}-\left(a+b\right)/2\right)},$$

let $h={l/l}^{*}$, then Equation (9) can be reshaped as Equation (10), then the point $x^{*}$ can be derived by applying Equation (10):

(10) $$x^{*}=\frac{a+b}{2}+\frac{a+b}{2h\eta }-\frac{x}{h\eta },$$

if h=1 and η=1, the Equation (10) can be changed to Equation (11) [37]:

(11) $$x^{*}=a+b-x,$$

In general, Equation (10) could be modified to handle D dimensional space problems, η is usually taken as 1, then it gets the following formula:

(12) $$x_{i,j}^{*}=\frac{a_{j}+b_{j}}{2}+\frac{a_{j}+b_{j}}{2h}-\frac{x_{i,j}}{h},j=1,2,\cdots ,D,$$

in which $x_{i,j}$ is the point of the $j^{th}$ dimension of the $i^{th}$ individual. The $x_{i,j}^{*}$ is the opposite position of $x_{i,j}.$ The $a_{j}$ and $b_{j}$ are the lower and upper bounds of the $j^{th}$ dimension on the search space, respectively.

Obviously, the solution obtained by using Equation (11) is fixed, and the changing refracted solution can be obtained by adjusting h in Equation (12), which further avoids the locally extreme value space.

### 3.3. ML Strategy

Generally, in each generation, the individual having the best function value is considered to be the optimal current generation individual. Nevertheless, greater knowledge in some dimensions may be provided by the individuals having worse fitness values. Hence, to facilitate the exchange of knowledge, individuals should improve their knowledge with the help of their interaction with each other. Given this, the ML strategy is motivated [32,38].

Let x be a point in [a,b], the ML individual can be obtained using Equation (13):

(13) $$x_{i,g}^{{}^{ML}}=x_{i,g}+\phi_{i,g}\left(x_{r,g}-x_{i,g}\right)|r\neq i,i=1,2,\ldots ,N_{p},$$

where $x_{r,g}$ denotes randomly chosen individual, and $\phi_{i,g}$ are a random number in [0,1]. 

### 3.4. ROML with Adjustment Factor Mechanism Strategy 

Let $X\in [L_{g}^{l\mathrm{ower}},L_{g}^{upper}]$ be a position in a D dimensional space in each generation g, where the bound vectors $L_{g}^{upper}=\left(L_{1,g}^{upper},L_{2,g}^{upper},\cdots ,L_{D,g}^{upper}\right)$ and $L_{g}^{lower}=\left(L_{1,g}^{lower},L_{2,g}^{lower},\cdots ,L_{D,g}^{lower}\right)$ are updated as:

(14) $$L_{j,g}^{upper}=max\left(\left[x_{1,j},x_{2,j},\cdots ,x_{N_{p},j}\right]\right),$$

(15) $$L_{j,g}^{lower}=min\left(\left[x_{1,j},x_{2,j},\cdots ,x_{N_{p},j}\right]\right),$$

where j=1,⋯,D. Define $C_{i,g}={\left[x_{1i,g},x_{2i,g},\ldots ,x_{Di,g}\right]}^{T}$ as a ROML individual of the present generation g, it can be given by applying Equation (16):

(16) $$C_{i,g}=\left\{ \begin{array}{l} \frac{L_{i,g}^{lower}+L_{i,g}^{upper}}{2}+\frac{L_{i,g}^{lower}+L_{i,g}^{upper}}{2h_{i}(g)}-\frac{X_{i,g}}{h_{i}(g)},\;if\;rand(0,1)<0.5,\\ \;X_{i,g}+\phi_{i,g}\left(X_{r,g}-X_{i,g}\right),\;otherwise, \end{array}\right.$$

in which $\phi_{i,g}$ is a random value of [0,1], $X_{r,g}(r\neq i)$ indicates a randomly chosen individual. A ROML population $P_{g}^{C}$ can be generated, i.e., $P_{g}^{C}=$$[C_{1,g},$$\ldots ,C_{N_{p},g}].$ Moreover, to keep ROML effective, the boundaries should be checked using Equation (17):

(17) $$C_{(i,j),g}=rand\left(L_{j,g}^{lower},L_{j,g}^{upper}\right),\;if\;C_{(i,j),g}<L_{j,g}^{lower}∥C_{(i,j),g}>L_{j,g}^{upper}.$$

After the ROML step, $N_{p}$ most suitable individuals are chosen from $\left\{P_{0},P_{g}^{C}\right\}$ according to their fitness values. Furthermore, it is worth noting that the moderator adjustment factor $h_{i}(FES)$ is an essential parameter affecting the learning performance of ROML. To achieve a wide range of refracted inverse solutions generated in the beginning stage of the algorithm and a small range of refracted opposite solutions generated in the later stage, a tuning factor that can be changed with the amount of function evaluation is designed by the trial-and-error method based on the literature [39] as follows:

(18) $$h_{i}(FES)={\left[1+{\left(FES/Max_{FES}\right)}^{\frac{1}{3}}\right]}^{15},$$

where FES are current function evaluations and maximal function evaluations are $Max_{FES}$. 

### 3.5. Proposed BROMLDE

In this section, the proposed BROMLDE with Bernstein operator, ROL strategy having adjustment factor mechanism, and ML strategy is presented in detail. First, the ROML population initialization procedure is presented. Then, the mutation and crossover with the Bernstein polynomials process are described. Finally, the ROML new population with generation jumping is stated, as well as the overall procedure of the proposed BROMLDE also be given.

#### 3.5.1. ROML Initialization

During the starting generation (g=0), the original population $P_{g}=P_{0}=[X_{1,0},\cdots ,X_{Np,0}]$ is generated using Equation (1). The ROML strategy is utilized to generate a new initial population $P_{0}^{C}=[C_{1,0},C_{2,0},\cdots ,C_{Np,0}]$, where $N_{p}$ is the population $P_{0}^{C}$ size, $C_{i,0},(i=1,\ldots ,N_{p})$ are obtained from Equation (16). Then, a new population is constituted via Equation (19):

(19) $$P_{0}^{*}=\left\{P_{0}\cup P_{0}^{C}\right\}.$$

The function values for ascending sorting of $P_{0}^{*}$ are computed by Equation (20):

(20) $$fitnessP_{0}^{*}=sort\left(ℱ\left(P_{0}^{*}\right)\right),$$

where ℱ indicates the objective function. Then, $N_{p}$ fittest individuals are picked from $P_{0}^{*}$ by using Equation (21). Moreover, to facilitate later understanding, we set $P_{\lambda ,g}=P_{\lambda ,0}.$

(21) $$\left[fitnessP_{\lambda ,0},P_{\lambda ,0}\right]=\left[fitnessP_{0}^{*},P_{0}^{*}\right]|\lambda \in \left[1:N_{p}\right].$$

Both the best solution, Pbest, and the global minimization function value, Psol, of the problem are calculated using Equation (22):

(22) $$\left[Psol,Pbest\right]=\left[min\left(fitnessP_{0}\right),P_{0}\right].$$

#### 3.5.2. Mutation and Crossover with Bernstein Polynomials

BROMLDE, which updates the starting mutation matrix $M_{(i,j),g}=0,i\in [1:N_{p}],$ j∈[1:D] at each iteration by utilizing Equation (23), controls the mutation process using the updated $M_{g}$.

(23) $$M_{(i,J),g}=1.$$

In Equation (23), J=u(⌈1:κ⋅D⌉)|u=permute(1:D) where the function permute(⋅) can arbitrarily change the sequence of the elements of (⋅). κ is given using Equation (24):

(24) $$\left\{ \begin{array}{l} switch\vartheta_{0}\\ \begin{array}{ll} \;case\;1\; & \kappa ={\left(1-\mu \right)}^{2},\\ \;case\;2\; & \kappa =2\cdot \mu \cdot \left(1-\mu \right),\\ \;case\;3\; & \kappa =\mu^{2}, \end{array}\\ end \end{array}\right.$$

where μ~U(0,1) and $\vartheta_{0}=[3\cdot \vartheta_{1}^{3}]$, $\vartheta_{1}\sim U[0\;1]$, $\vartheta_{0}\in U\left\{1:3\right\}$, the κ is computed employing $2^{nd}$ degree Bernstein polynomials. The step size $F_{g}$ of the evolution is obtained by using Equation (25):

(25) $$F_{g}=\left\{ \begin{array}{l} {\left({\left[\xi_{(1,1:D),g}^{3}\circ \left|\gamma_{(1,1:D),g}^{3}\right|\right]}^{\prime }\times Q_{(1,1:N_{p}),g}\right)}^{\prime },\;if\;\vartheta_{2}<\vartheta_{3},\\ \gamma_{(N_{p},1),g}^{3}\times Q_{(1,D),g},\;otherwise, \end{array}\right.$$

where $\vartheta_{\left(2:3\right)},\xi_{g}$ and $\gamma_{g}$ are random values that will be updated with each call, where $\vartheta_{(2:3),g},\xi_{g}\sim U(0,1),\gamma_{g}\sim N(0,1)$, and matrix $Q_{(\cdot ,\cdot ),g}=1$.

In BROMLDE, the trial vector $T_{g}$ is obtained by making use of Equation (26):

(26) $$T_{g}=F_{g}\circ M_{g}\circ \left({\left(w^{*}\right)}^{3}\circ E_{g}+\left(1-{\left(w^{*}\right)}^{3}\right)\circ \;Pbest-P_{g}\right)+P_{g}|w_{\left(1:N_{p},1\right)}^{*}\sim U(0,1),$$

where $E_{g}=w\circ P_{K_{1},g}+(1-w)\circ P_{K_{2},g}|w_{(1:N_{p},1:D),g}\sim U(0,1)$, ∘ indicates Hadamart multiplication operator, $K_{1}$ and $K_{2}$ are specified in Equation (27):

(27) $$K_{1}=permute\left(1:N_{p}\right),K_{2}=permute\left(1:N_{p}\right)|K_{1}\neq \left[1:N_{p}\right],K_{1}\neq K_{2}.$$

If $T_{(i,j),g}<low_{j,g}||T_{(i,j),g}>up_{j,g}$, $T_{(i,j),g}$ values are updated using the Equation (28):

(28) $$T_{(i,j),g}=\left\{ \begin{array}{l} low_{j,g}+\alpha^{3}\left(up_{j,g}-low_{j,g}\right),\;if\;T_{(i,j),g}<low_{j,g},\\ up_{j,g}+\alpha^{3}\left(low_{j,g}-up_{j,g}\right),\;if\;T_{(i,j),g}>up_{j,g}, \end{array}\right.$$

where α~U(0,1). The function values of $T_{g}$ are obtained by applying Equation (29):

(29) $$fitnessT_{g}=ℱ\left(T_{g}\right).$$

Based on the selection process of Equation (30), an updated population can be obtained.

(30) $$if\;fitnessT_{\lambda ,g}<fitnessP_{\lambda ,g},\left[P_{\lambda ,g},fitnessP_{\lambda ,g}\right]=[T_{\lambda ,g},fitnessT_{\lambda ,g}]|\lambda \in \left[1:N\right].$$

#### 3.5.3. ROML New Population with Generation Jumping

Now, based on the updated population $P_{g}$ in Equation (30), a new ROML population $P_{g}^{C}=[C_{1,g},C_{2,g},\cdots ,C_{Np,g}]$ can be obtained by Equations (16)–(18), if the jumping rate $J_{r}$ is bigger than the selection probability, where $N_{p}$ is the population $P_{g}^{C}$ size. The objective function values of the new population $P_{g}^{C}$ are computed by Equation (31):

(31) $$fitnessP_{g}^{C}=ℱ\left(P_{g}^{C}\right).$$

Then, the new population $P_{g}^{*}$, including $P_{g}$ and $P_{g}^{C}$, and its objective function values for ascending sorting are denoted as follows:

(32) $$\left\{ \begin{array}{l} P_{g}^{*}=\left\{P_{g}\cup P_{g}^{C}\right\}\\ fitnessP_{g}^{*}=sort\left\{fitnessP_{g}\cup fitnessP_{g}^{C}\right\}. \end{array}\right.$$

Afterward, $N_{p}$ most suitable individuals are selected from $P_{g}^{*}$ using Equation (33):

(33) $$\left[\;fitnessP_{\lambda ,g},P_{\lambda ,g}\right]=\left[fitnessP_{g}^{*},P_{g}^{*}\right]|\lambda \in \left[1:N_{p}\right].$$

Based on Equations (31)–(33), the individuals $P_{\lambda ,g},\lambda \in [1:N_{p}]$ obtaining a better objective function value will make up the new generation population.

In the current evolutionary step, both the optimal solution, Pbest, and corresponding objective function value, Psol, are provided by the updated population $P_{g}$ in Equation (33), and both of them are updated by employing Equation (34):

(34) $$\left[Psol,Pbest\right]=\left[min\left(fitnessP_{g}\right),P_{g}\right].$$

The pseudo-code of BROMLDE is provided in Algorithm 1.
**Algorithm 1:** The BROMLDE Algorithm Procedure   **Input:** Objective function:ℱ, Search-space limits: (low,up), Population size: $N_{p}$,      Dimension of problem:D, Maximal function evaluations: $Max_{FES},$ Jumping      rate: $J_{r}$   **Output:** Psol: Global minimum Pbest: Global minimizer 1  Set the current function evaluations FES=0 2  Set the population bounds randomly generate an initial population $P_{0}$ 3  **for**
i=1
**to**
$N_{p}$
**do**    //ROML population initialization (generation g = 0) 4    $\phi_{i,0}=rand(0,1)$ 5    $h_{i}(FES)={\left(1+\sqrt[{3}]{\frac{FES}{Max_{FES}}}\right)}^{15}$ 6    $C_{i,0}=\left\{ \begin{array}{l} \frac{L_{i,0}^{lower}+L_{i,0}^{upper}}{2}+\frac{L_{i,0}^{lower}+L_{i,0}^{upper}}{2h_{i}(g)}-\frac{X_{i,0}}{h_{i}(g)},\;if\;rand(0,1)<0.5,\\ \;X_{i,0}+\phi_{i,0}(X_{r,0}-X_{i,0}),\;otherwise, \end{array}\right.$ 7   Check the bounds in the current generation by Equation (17) 8  **end** 9  Get population $P_{g}$ by selecting $N_{p}$ fittest points from $\left\{P_{0}\cup P_{0}^{C}\right\}$ 10  $FES=FES+2N_{p}$ 11  Get Psol and Pbest by Equations (20)–(22) 12  **while**
$FES<Max_{FES}$ **do  //**Main loop(generation g > 0) 13   $M_{(i,j),g}=0$   **//**Generate mutation matrix (M) 14   **for**
i=1
**to**
$N_{p}$
**do** 15     u=permute(1:D) 16     Generate μ, where μ~U(0,1) 17     Generate $\vartheta_{0},\vartheta_{0}=[3\cdot \vartheta_{1}^{3}],\vartheta_{1}\sim U[0\;1],\vartheta_{0}\in U\{1:3\}$ 18     **switch** $\vartheta_{0}$ **do** 19       $\mathrm{case}\;1\;\mathrm{do}\;\kappa ={\left(1-\mu \right)}^{2}$; 20       case 2 do κ=2⋅μ⋅(1−μ); 21       $\mathrm{case}\;3\;\mathrm{do}\;\kappa =\mu^{2};$ 22     **end** 23     J=u(⌈1:κ⋅D⌉)|u=permute(1:D); $M_{(i,J),g}=1$ 24   **end** 25   Calculate the evolutionary step size $F_{g}$ by Equation (25) 26   Generate the trial vector $T_{g}$ by Equations (26) and (27) and control the boundaries of $T_{g}$ by Equation (28) 27   Update population $P_{g}$ by Equations (29) and (30) 28   $FES=FES+N_{p}$ 29   **if** $rand\leq J_{r}$ **then**  //ROML population with generation jumping 30    Update the bounds by calculating the smallest and biggest values of all     dimensions in the population $P_{g}$ 31    **for** i=1
**to**
$N_{p}$
**do** 32     $\phi_{i,g}=rand(0,1)$ 33     $h_{i}(FES)={\left(1+\sqrt[{3}]{\frac{FES}{Max_{FES}}}\right)}^{15}$ 34     $C_{i,g}=\left\{ \begin{array}{l} \frac{L_{i,g}^{lower}+L_{i,g}^{upper}}{2}+\frac{L_{i,g}^{lower}+L_{i,g}^{upper}}{2h_{i}(g)}-\frac{X_{i,g}}{h_{i}(g)},\;if\;rand(0,1)<0.5,\\ \;X_{i,g}+\phi_{i,g}(X_{r,g}-X_{i,g}),\;otherwise, \end{array}\right.$ 35     Check the bounds in the current generation by Equation (17) 36    **end** 37    Select $N_{p}$ fittest individuals from $\left\{P_{g}\cup P_{g}^{C}\right\}$ and update the population $P_{g}$     by Equation (33) 38    $FES=FES+N_{p}$ 39   **end** 40   Update Psol and Pbest by Equation (34)
41  **end**

### 3.6. Computational Complexity

The computational complexity of BROMLDE mainly depends on three parts: ROML population initialization, mutation, crossover, and selection, as well as ROML new population with generation jumping. The complexity of these worst-case scenarios is as below:

(1) For the ROML population initialization, the process requires generating the starting population and its corresponding RMOL population and then selecting the $N_{p}$ best individuals from the two populations as the new initial population of the algorithm. Therefore, the time complexity is $O(Np\cdot D)+O\left(Np\cdot \log_{2}(2Np)\right).$
(2) In the mutation, crossover, and selection of the BROMLDE algorithm, the algorithm mainly includes the initialization and update of starting mutation matrix M, the obtaining of step size $F_{g}$, and trial vector $T_{g}$. The time complexity is O(Np⋅D)+O(Np)+O(D).
(3) In ROML new population with generation jumping, it consists mainly of the generation of the ROML population with $N_{p}$ size, and the selection of $N_{p}$ most suitable individuals from the ROML population and initial population. Further, the time complexity is $O(Np\cdot D)+O\left(Np\cdot \log_{2}(2Np)\right).$

Thus, the whole-time complexity of the developed BROMLDE can be estimated as $O(Np\cdot D)+O(Np)+O(D)+O\left(Np\cdot \log_{2}(2Np)\right)$.

## 4. Numerical Experiments and Results Analysis

### 4.1. Experiment Setup

To investigate the performance of the developed BROMLDE, numerical experiments on the CEC 2019 benchmark functions [40] (see Table 1) and CEC 2020 test suites [41] (see Table 2) are conducted by comparison with various well-known optimization methods. Those methods include BSDE [19], OMLDE [32], weighted differential evolution (WDE) [42], adaptive DE with optional external archive (JADE) [43], success-history adaptation DE (SHADE) [44], PSO [8], CMAES [45], and simulated annealing (SA) [9]. Moreover, BROMLDE is also compared to the IEEE CEC 2020 winning DE algorithm variants IMODE [46] and J2020 [47] to evaluate its performance. To make the experimental comparison fair, the comparison algorithm is run under identical test conditions. The whole numerical experiments are performed on PlatEMO [48] of MATLAB 2021b on a computer with CPU AMD Ryzen 5 3550H @2.10GHz and 16G RAM, Win10 64-bit operating system. The population size $N_{p}$ is 100, and the jumping rate $J_{r}$ is 0.05, which is consistent with OMLDE. Then, the maximal function evaluations ($Max_{FES}$) are set to 10,000 as the termination condition, and 30 independent runs are performed. Moreover, the parameter settings of other counterparts refer to their settings.

Moreover, the Wilcoxon rank-sum test with a significant level of α=0.05 is employed to judge the difference between BROMLDE and its competitors in this paper. More specifically, the results of taking the minimum fitness function value for each of the 30 independent runs are obtained. Then, the probability *p*-value corresponding to the BROMLDE algorithm and each of its competitors is calculated separately by using MATLAB. Finally, the determination of whether there are significant differences between algorithms is based on the *p*-value and significance level α. The symbols applied to the Wilcoxon rank-sum test are described as “+”, ”−”, and “=”, which indicate that BROMLDE has significantly superior, inferior, and no significant difference between BROMLDE and the compared algorithm, respectively. Furthermore, the basic statistical evaluations including the global minimum average (AVG) and global minimum standard deviation (STD) are utilized for the obtained minimum fitness value results.

### 4.2. Numerical Function Optimization Problems

#### 4.2.1. Experimental Results for CEC 2019

This subsection focuses on comparing the optimization results of BROMLDE and other methods including BSDE, OMLDE, WDE, PSO, SHADE, JADE, and CMAES to solve the CEC 2019 benchmark functions (see Table 1). The AVG and STD of the results obtained from the tests performed using F1–F10 are listed in Table 3. The minimum AVG in Table 3 is highlighted in bold. Based on the results of the minimum fitness value, the Wilcoxon rank-sum test findings of BROMLDE and its competitors in Table 4 are symbolized (+, −, =). +, =, and − indicate that BROMLDE performs better, equal, and worse than the compared methods, respectively.

As presented in Table 1, CEC 2019 benchmark functions have different dimensions, the dimensions of the functions F1, F2, and F3 are 9, 16, and 18, respectively. In addition, functions F4–F10 have the same dimension 10. According to the experimental setting rules in Section 4.1, the AVG and STD of the minimum fitness values on F1–F10 of the CEC 2019 benchmark functions are recorded in Table 3, we can see that BROMLDE obtains the minimum AVG for 6 functions out of the total 10 functions compared to the other algorithms, which are functions F1, F3, F6, F7, F8, and F10. 

According to the Wilcoxon rank-sum test results in Table 4 (last line), we can see that BROMLDE achieves more than 6 significantly better results (“+”) compared to BSDE, OMLDE, WDE, SHADE, and CMAES; BROMLDE also yields more than 5 superior results compared to PSO and JADE. In other words, the mean percentage of the goodness of BROMLDE for the 10 functions is 72.86% $\left(\sum_{i=1}^{7}{+}_{i}/\left(10\times 7\right)\times 100\%\right)$. The general results show that the ROML strategy can effectively enhance the optimization ability of DE.

**Figure 2:**
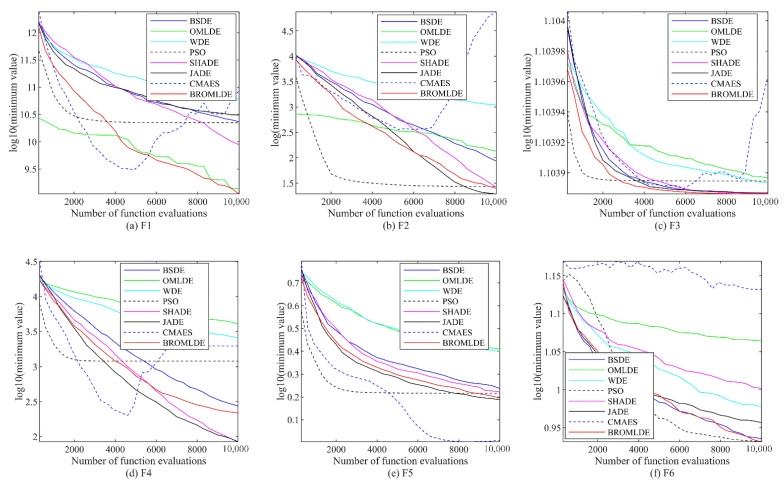

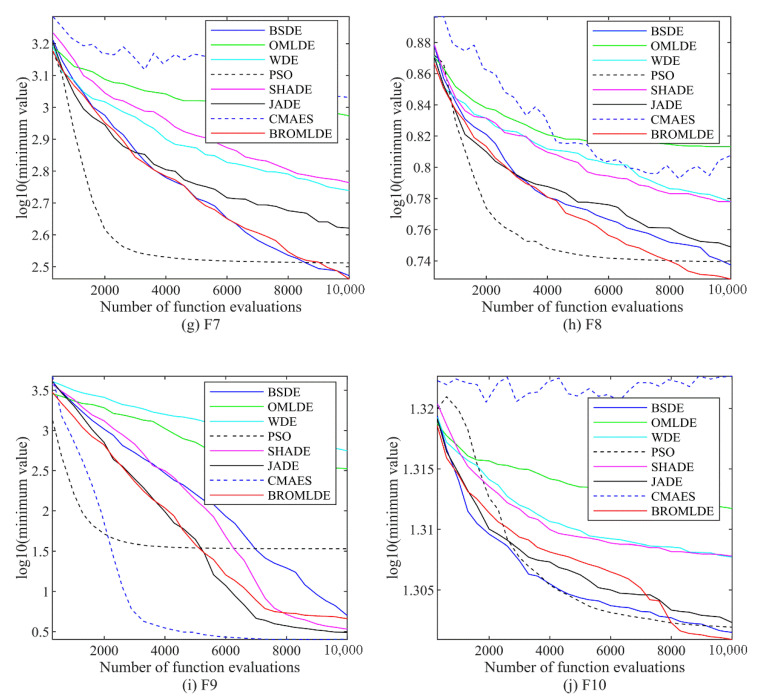
Convergence graph of BROMLDE and its competitors on CEC 2019.

Figure 2 presents the convergence diagrams of BROMLDE and other methods for the 10 tested functions (F1–F10) on CEC 2019, where the vertical axis is the logarithm of the minimum value of the functions and the horizontal axis is functional evaluation numbers. It can be seen that although PSO converges fastest, the local optimum situation occurs. BROMLDE has a fast descent rate on most of the tested functions. Moreover, we can also conclude that for a limited number of evaluations, smaller fitness values can be obtained for BROMLDE on the functions F1, F3, F6, F7, F8, and F10. The good performance of BROMLDE is due to the initialization of ROML at the start and the exploring ability of ROML.

#### 4.2.2. Experimental Results for CEC 2020

The optimization results of BROMLDE and other algorithms, including BSDE [19], OMLDE [32], PSO, SA, IMODE [46], and J2020 [47], to solve the CEC 2020 benchmark functions in dimensions 5 and 10 are compared in this subsection.

As shown in Table 2, those benchmark functions can be generally divided into the unimodal function (F1), multimodal shifted and rotated functions (F2–F4), hybrid functions (F5–F7), and composition functions (F8–F10). Based on the experimental setting rules in Section 4.1, the AVG, STD and Wilcoxon rank-sum test results of the test functions derived from the proposed BROMLDE and other test approaches are calculated by using F1–F10 when the problem dimension D is equal to 5 and 10 are displayed in Table 5, Table 6, Table 7 and Table 8. The smallest average of each function in the table is marked in bold font. Additionally, according to the statistical results, the Wilcoxon rank-sum test is employed to determine the differences between BROMLDE and its competitors (+, =, and − denote that BROMLDE performs better, equal, and worse than its competitors, respectively). 

For the 5-dimensional test problems, based on Table 5, one can see that BROMLDE exhibits outstanding performance compared to other tested methods. Among the 10 CEC 2020 test functions, BROMLDE yields five minimum fitness value results, namely two multimodal shifted and rotated functions F3 and F4, two hybrid functions F5 and F6, and one composition function F9. This shows that BROMLDE is more dominant in solving multimodal and hybrid function problems than the compared algorithm. Furthermore, from Table 6 (last line), BROMLDE is superior to BSDE, OMLDE, WDE, PSO, SA, J2020, and IMODE by 7, 10, 10, 6, 9, 5, and 4 cases, respectively, among the 10 functions. At this time, the mean good rate of BROMLDE reaches 72.86% $\left(\left(\sum_{i=1}^{7}{+}_{i}\right)/\left(10\times 7\right)\times 100\%\right)$. That is to say, those results significantly exceed the inferior functions. 

BROMLDE still shows excellent performance among all these methods when the dimension of the problem is increased to 10. The results are provided in Table 7 and Table 8. In detail, the developed BROMLDE is still the winner compared to OMLDE, WDE, PSO, SA, and IMODE on more than 5 functions based on the Wilcoxon rank-sum test findings in Table 8 (last line). The percentage of the goodness of BROMLDE on 10 functions is 77.14%. For the AVG and STD of the fitness values in Table 7, we can see that BROMLDE also maintains the highest ranking with more than 4 best results. Meanwhile, BROMLDE still has great potential in solving hybrid function problems. Based on the analysis of the above findings, we can summarize that BROMLDE performs excellently compared to other tested algorithms.

**Figure 3:**
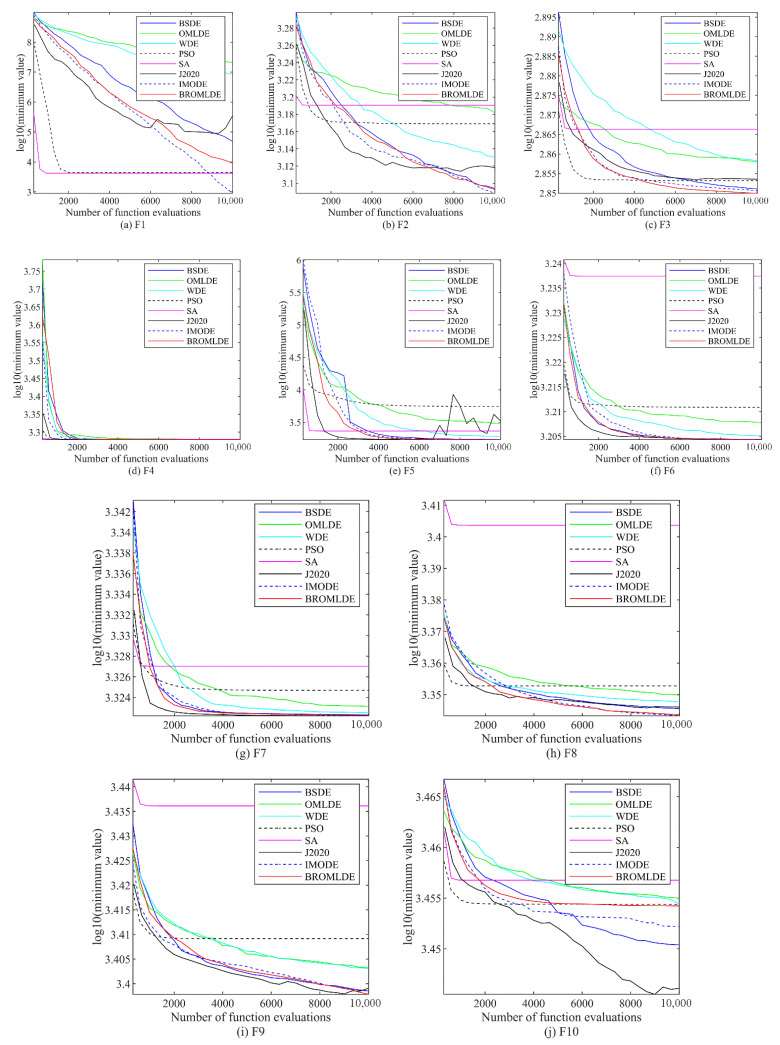
Convergence graphs of F1–F10 in 5D.

**Figure 4:**
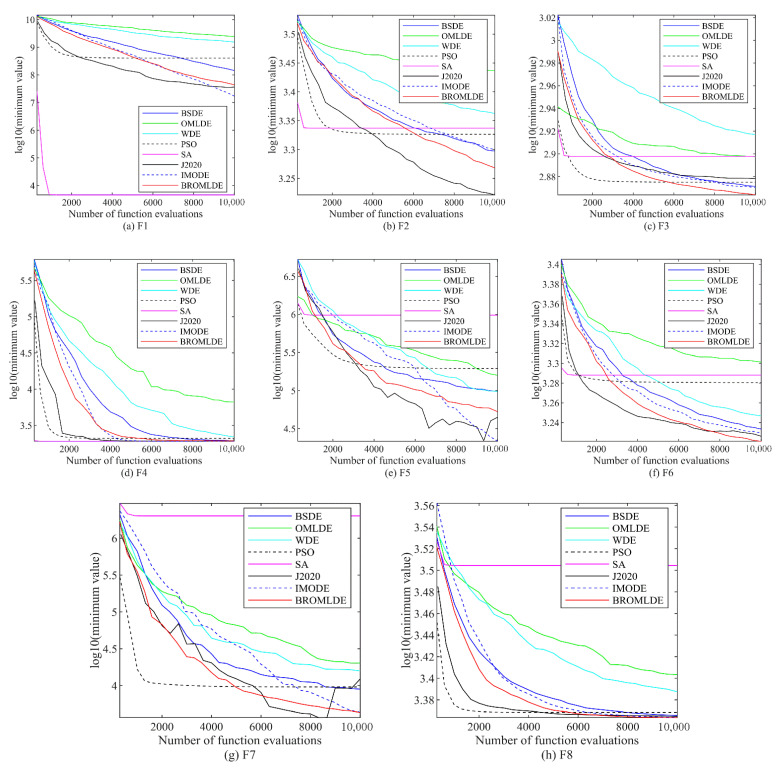

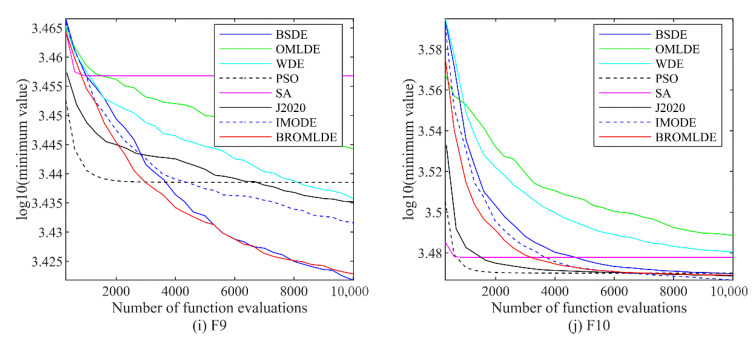
Convergence graphs of F1–F10 in 10D.

The convergence plots of BROMLDE and other compared approaches on the 5 and 10-dimensional CEC 2020 functions (F1–F10) are presented in Figure 3 and Figure 4, respectively. In those plots, the vertical axis is the logarithm of the minimum value of the functions and the horizontal axis is the functional evaluation numbers. From those figures, we can see that although PSO and SA converge faster on some functions, the local optimum situation may occur. Compared with other algorithms, BROMLDE has a faster descent speed and better optimization capability in most functions. The reason is that the combination of the Bernstein search and the ROML strategy allows BROMLDE to reach a better trade-off between global exploration and local exploitation capabilities. In the early stage, the ROML strategy can provide strong search abilities and helps to localize the exact search in the late stage. Moreover, Bernstein search may reduce the difficulty of parameter setting and improve the convergence accuracy. It reveals that our proposed BROMLDE can reach better convergence properties and global optimization ability.

#### 4.2.3. Comparison with IMODE

In this subsection, on the basis of Section 4.1, the 20-dimensional CEC 2020 test functions (F1–F10) are used to compare the performance of BROMLDE and IMODE, the AVG and STD results are presented in Table 9, and the Wilcoxon rank-sum test findings are also embedded in it. One can see that IMODE has a greater advantage in solving unimodal function (F1) and multimodal shifted and rotated functions except for F3. BROMLDE is superior to IMODE in solving hybrid functions F5–F7. In addition, BROMLDE outperforms IMODE in solving the composition functions F8 and F10 and is worse in solving F9 of composition functions. The Wilcoxon rank-sum test results (+, =, and − denote that BROMLDE performs better, equal, and worse than IMODE, respectively) display that the average good rate of BROMLDE is 25% ($\frac{\mathrm{statistics}\;\mathrm{number}:+}{4}\times 100\%$) for the unimodal function and the multimodal functions, is 66.67% ($\frac{\mathrm{statistics}\;\mathrm{number}:+}{3}\times 100\%$) for hybrid functions, and is 33.33% ($\frac{\mathrm{statistics}\;\mathrm{number}:+}{3}\times 100\%$) for composition functions. Those results also reflect the no free lunch theorem that no single algorithm can be applied to all optimization problems [33]. Based on the above analysis, one can conclude that BROMLDE is more appropriate for solving hybrid function problems and it performs worse in solving unimodal functions and the multimodal shifted and rotated functions as well as composition functions.

### 4.3. Real-World Engineering Optimization Problems

To further verify the feasibility of our proposed BROMLDE in practical engineering applications, for the solution of the car side impact (CSI) design problem and the speed reducer (SR) design problem, BROMLDE, some DE variants (BSDE, OMLDE, and WDE) and the superior algorithms (PSO and SNS) for solving these problems are used.

#### 4.3.1. CSI Design Problem

**Figure 5:**
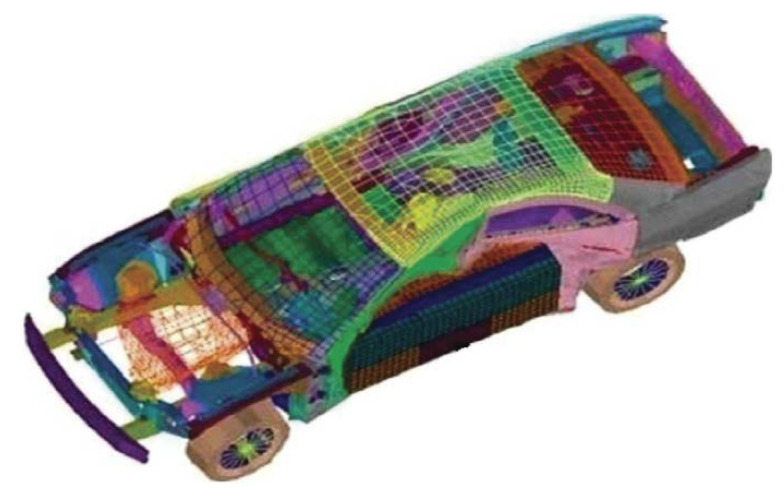
A model of CSI design problem [6].

The goal of the CSI design problem is to obtain the minimum weight of the door satisfying 10 constraints on 11 influence variables [6]. Those variables are listed in Table 10. The authors in [49] simplify the analytical formulation of this optimization problem. Figure 5 [6] shows a model for the CSI design problem. Then, the objective function of this design problem is Equation (35):

(35) $$min\;f(x)=1.98+4.90x_{1}+6.67x_{2}+6.98x_{3}+4.01x_{4}+1.78x_{5}+2.73x_{7}$$

subject to:

(36) $$\left\{ \begin{array}{ll} \Phi_{1}(x) & =\;1.16-0.3717x_{2}x_{4}-0.00931x_{2}x_{10}-0.484x_{3}x_{9}+0.01343x_{6}x_{10}-1\leq 0,\\ \Phi_{2}(x) & =\;46.36-9.9x_{2}-12.9x_{1}x_{2}+0.1107x_{3}x_{10}-32\leq 0,\\ \Phi_{3}(x) & =\;33.86+2.95x_{3}+0.1792x_{3}-5.057x_{1}x_{2}-11.0x_{2}x_{8}-0.0215x_{5}x_{10}-9.98x_{7}x_{8}\\ & +\;22.0x_{8}x_{9}-32\leq 0,\\ \Phi_{4}(x) & =\;28.98+3.818x_{3}-4.2x_{1}x_{2}+0.0207x_{5}x_{10}+6.63x_{6}x_{9}-7.7x_{7}x_{8}+0.32x_{9}x_{10}-32\leq 0,\\ \Phi_{5}(x) & =\;0.261-0.0159x_{1}x_{2}-0.188x_{1}x_{8}-0.019x_{2}x_{7}+0.0144x_{3}x_{5}+0.0008757x_{5}x_{10}\\ & +\;0.08045x_{6}x_{9}+0.00139x_{8}x_{11}+0.00001575x_{10}x_{11}-0.32\leq 0,\\ \Phi_{6}(x) & =\;0.214+0.00817x_{5}-0.131x_{1}x_{8}-0.0704x_{1}x_{9}+0.03099x_{2}x_{6}-0.018x_{2}x_{7}+0.0208x_{3}x_{8}\\ & +\;0.121x_{3}x_{9}-0.00364x_{5}x_{6}+0.0007715x_{5}x_{10}-0.0005354x_{6}x_{10}+0.00121x_{8}x_{11}\\ & +\;0.00184x_{9}x_{10}-0.02x_{2}^{2}-0.32\leq 0,\\ \Phi_{7}(x) & =\;0.74-0.61x_{2}-0.163x_{3}x_{8}+0.001232x_{3}x_{10}-0.166x_{7}x_{9}+0.227x_{2}^{2}-0.32\leq 0,\\ \Phi_{8}(x) & =\;4.72-0.5x_{4}-0.19x_{2}x_{3}-0.0122x_{4}x_{10}+0.009325x_{6}x_{10}+0.000191x_{11}^{2}-4\leq 0,\\ \Phi_{9}(x) & =\;10.58-0.674x_{1}x_{2}-1.95x_{2}x_{8}+0.02054x_{3}x_{10}-0.0198x_{4}x_{10}+0.028x_{6}x_{10}-9.9\leq 0,\\ \Phi_{10}(x) & =\;16.45-0.489x_{3}x_{7}-0.843x_{5}x_{6}+0.0432x_{9}x_{10}-0.0556x_{9}x_{11}-0.000786x_{11}^{2}-15.7\leq 0, \end{array}\right.$$

in which $0.5\leq x_{i}\leq 1.5,i=1,\ldots ,7,x_{8},x_{9}\in \left\{0.192,0.345\right\}$ and $-30\leq x_{10},x_{11}\leq 30.$

Table 11 concludes the best and workable experimental results of BROMLDE and its competitors after 30 independent runs (where $N_{p}=100,Max_{FES}=15,000$). From Table 11, BROMLDE can get the optimal objective function value, i.e., 22.2372. Furthermore, our proposed BROMLDE has the smallest AVG and STD, and these are 2.2463 × 10 and 1.4463 × 10^−1^, respectively, compared to other algorithms tested here. Moreover, based on the Wilcoxon rank-sum test results (+, =, and − denote that BROMLDE performs better, equal, and worse than the compared algorithm, respectively), we can obtain that BROMLDE is better than BSDE, OMLDE, WDE, PSO, and SNS, respectively. It can be concluded that the effectiveness and reliability of the proposed BROMLDE are superior to other tested algorithms.

#### 4.3.2. SR Design Problem

**Figure 6:**
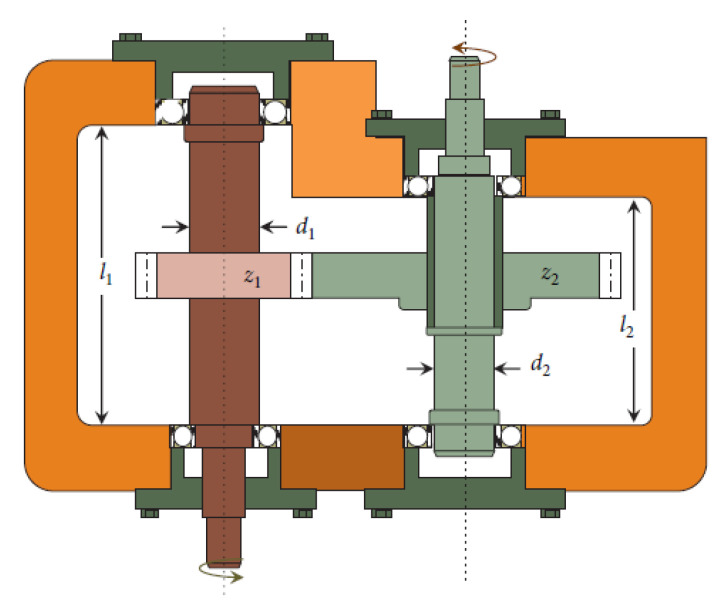
Schematic diagram of the SR [6].

SR design problem (see Figure 6) aims to design the speed reducer subject to 11 constraints. One has 7 variables (see Table 12). The mathematical expression of this problem is given in Equation (37) [6]:

(37) $$\begin{array}{cc} min\;f(x) & =\;0.7854\cdot x_{1}\cdot x_{2}^{2}\cdot \left(3.3333\cdot x_{3}^{2}+14.9334\cdot x_{3}-43.0934\right)-1.508\cdot x_{1}\cdot \left(x_{6}^{2}+x_{7}^{2}\right)\\ & +\;7.4777\cdot \left(x_{6}^{2}+x_{7}^{2}\right)+0.78054\cdot \left(x_{4}\cdot x_{6}^{2}+x_{5}\cdot x_{7}^{2}\right), \end{array}$$

subject to:

(38) $$\begin{array}{l} \Phi_{1}(x)=\frac{27}{x_{1}\cdot x_{2}^{2}\cdot x_{3}}-1\leq 0,\\ \Phi_{2}(x)=\frac{397.5}{x_{1}\cdot x_{2}^{2}\cdot x_{3}^{2}}-1\leq 0,\\ \Phi_{3}(x)=\frac{1.93\cdot x_{4}^{3}}{x_{2}\cdot x_{3}\cdot x_{6}^{4}}-1\leq 0,\\ \Phi_{4}(x)=\frac{1.93\cdot x_{5}^{3}}{x_{2}\cdot x_{3}\cdot x_{7}^{4}}-1\leq 0,\\ \Phi_{5}(x)=\sqrt{{\left(\frac{745.0\cdot x_{4}}{x_{2}\cdot x_{3}}\right)}^{2}+16.9\times 10^{6}}/(110x_{6}^{3})-1\leq 0,\\ \Phi_{6}(x)=\sqrt{{\left(\frac{745.0\cdot x_{5}}{x_{2}\cdot x_{3}}\right)}^{2}+157.5\times 10^{6}}/\left(85x_{7}^{3}\right)-1\leq 0,\\ \Phi_{7}(x)=\frac{x_{2}\cdot x_{3}}{40}-1\leq 0,\\ \Phi_{8}(x)=\frac{5x_{2}}{x_{1}}-1\leq 0,\\ \Phi_{9}(x)=\frac{x_{1}}{12x_{2}}-1\leq 0,\\ \Phi_{10}(x)=\frac{1.5x_{6}+1.9}{x_{4}}-1\leq 0,\\ \Phi_{11}(x)=\frac{1.1x_{7}+1.9}{x_{5}}-1\leq 0, \end{array}$$

in which the bounds are as follows:

(39) $$\left\{ \begin{array}{l} 2.6\leq x_{1}\leq 3.6\\ 0.7\leq x_{2}\leq 0.8\\ 17\leq x_{3}\leq 28\\ 7.3\leq x_{4}\leq 8.3\\ 7.3\leq x_{5}\leq 8.3\\ 2.9\leq x_{6}\leq 3.9\\ 5.0\leq x_{7}\leq 5.5 \end{array}\right.$$

Table 13 records the optimal and feasible experimental results of BROMLDE and the tested algorithms after 30 independent runs (where $N_{p}=100,Max_{FES}=15,000$). According to Table 13, BROMLDE can obtain the optimal fitness value, i.e., 5.4421 × 10^3^. Meanwhile, the proposed BROMLDE has the minimum AVG (5.4660 × 10^3^) compared with other competitors. Through the Wilcoxon rank-sum test results (+, =, and − denote that BROMLDE performs better, equal, and worse than the compared algorithm, respectively), we can observe that BROMLDE is still better than most algorithms. This further shows that our proposed algorithm is workable in solving practical problems.

## 5. Conclusions and Future Work

In this paper, a DE algorithm based on the Bernstein operator and refracted oppositional-mutual learning strategy is proposed to enhance the optimization effect of the algorithm. More specifically, a random switching scheme allows the selection of ROL and ML for all individuals in the ROML initialization phase and the ROML generation jumping phase, which helps to balance the exploration and exploitation. A dynamic adjustment factor varying with the number of evaluations in the ROML strategy is proposed, contributing to the tuning of the search space and jumping out of the local optimum. Moreover, a Bernstein operator is introduced to control the mutation and crossover phases, improving the convergence accuracy of the algorithm, and making it more efficient. Experiments are performed on CEC 2019 and CEC 2020 benchmark functions, and the experimental results show that the proposed BROMLDE outperforms the compared algorithm. Meanwhile, the Wilcoxon rank-sum test and convergence analysis reveal that the BROMLDE is considerably better than other tested algorithms. Particularly, BROMLDE is superior to IMODE in solving hybrid function problems from CEC 2020 (20D). Additionally, BROMLDE and the tested algorithms (BSDE, OMLDE, WDE, PSO, and SNS) are used on a practical engineering problem, and the result further verifies the applicability of the algorithm in solving real-life engineering issues. Therefore, it is worth recommending ROML strategies to other algorithms to enhance their performance. However, when BROMLDE is compared with IMODE for the CEC 2020 test functions, BROMLDE is only superior in solving hybrid function problems and performs inferiorly in other problems. Given this, we will explore new learning strategies to further improve the performance of the algorithm in future research work.

## Figures and Tables

**Table 1:** CEC 2019 benchmark functions [40].

No.	Functions	*D*	Search Range	Best
F1	Storn’s Chebyshev Polynomial Fitting Problem	9	[−8192, 8192]	1
F2	Inverse Hilbert Matrix Problem	16	[−16,384, 16,384]	1
F3	Lennard–Jones Minimum Energy Cluster	18	[−4, 4]	1
F4	Rastrigin’s Function	10	[−100, 100]	1
F5	Griewangk’s Function	10	[−100, 100]	1
F6	Weierstrass Function	10	[−100, 100]	1
F7	Modified Schwefel’s Function	10	[−100, 100]	1
F8	Expanded Schaffer’s F6 Function	10	[−100, 100]	1
F9	Happy Cat Function	10	[−100, 100]	1
F10	Ackley Function	10	[−100, 100]	1

**Table 2:** CEC 2020 test suites [41].

	No.	Functions	Best
Unimodal Function	F1	Shifted and Rotated Bent Cigar Function	100
Multimodal Shifted and Rotated Functions	F2	Shifted and Rotated Schwefel’s Function	1100
	F3	Shifted and Rotated Lunacek bi-Rastrigin Function	700
	F4	Expanded Rosenbrock’s Plus Griewangk’s Function	1900
Hybrid Functions	F5	Hybrid Function 1 (N = 3)	1700
	F6	Hybrid Function 2 (N = 4)	1600
	F7	Hybrid Function 3 (N = 5)	2100
Composition Functions	F8	Composition Function 1 (N = 3)	2200
	F9	Composition Function 2 (N = 4)	2400
	F10	Composition Function 3 (N = 5)	2500
Search Range: ${\left[-100,100\right]}^{D}$(D is the population dimension)			

**Table 3:** Minimum fitness value on F1–F10 of CEC 2019 benchmark functions.

No.	Metric	BSDE	OMLDE	WDE	PSO	SHADE	JADE	CMAES	BROMLDE
F1	AVG	2.3365 × 10^10^	1.3686 × 10^9^	6.0428 × 10^10^	2.2371 × 10^10^	8.8385 × 10^9^	3.1163 × 10^10^	8.6816 × 10^10^	**1.1022 × 10^9^**
	STD	1.3624 × 10^10^	2.1840 × 10^9^	3.4700 × 10^10^	1.7275 × 10^10^	4.8209 × 10^9^	1.5759 × 10^10^	1.6158 × 10^11^	1.6019 × 10^9^
F2	AVG	8.6463 × 10	1.3452 × 10^2^	1.0931 × 10^3^	2.7232 × 10	2.6338 × 10	**1.9322 × 10**	7.5171 × 10^4^	2.5057 × 10
	STD	3.8929 × 10	1.2929 × 10^2^	4.4152 × 10^2^	5.3829 × 10	4.5771	2.7313	1.2393 × 10^4^	1.0313 × 10
F3	AVG	**1.2702 × 10**	1.2703 × 10	1.2703 × 10	1.2703 × 10	**1.2702 × 10**	**1.2702 × 10**	1.2705 × 10	**1.2702 × 10**
	STD	9.0900 × 10^−6^	2.0974 × 10^−4^	1.3621 × 10^−4^	7.5574 × 10^−4^	6.5941 × 10^−6^	1.9165 × 10^−5^	3.1064 × 10^−3^	4.5257 × 10^−6^
F4	AVG	2.7441 × 10^2^	4.0491 × 10^3^	2.5733 × 10^3^	1.2030 × 10^3^	8.4969 × 10	**8.4365 × 10**	1.9674 × 10^3^	2.1898 × 10^2^
	STD	7.4444 × 10	2.1316 × 10^3^	7.7972 × 10^2^	6.6790 × 10^2^	1.3287 × 10	2.5848 × 10	1.0480 × 10^4^	1.6873 × 10^2^
F5	AVG	1.7300	2.5679	2.5137	1.6473	1.6712	1.5414	**1.0218**	1.5682
	STD	1.3887 × 10^−1^	3.1217 × 10^−1^	1.8449 × 10^−1^	4.3138 × 10^−1^	1.0437 × 10^−1^	1.1315 × 10^−1^	1.1879 × 10^−1^	8.3493 × 10^−2^
F6	AVG	8.6195	1.1608 × 10	9.4992	8.5524	1.0043 × 10	9.0537	1.3562 × 10	**8.5392**
	STD	6.7997 × 10^−1^	6.7551 × 10^−1^	8.8662 × 10^−1^	1.1533	7.6363 × 10^−1^	6.5637 × 10^−1^	6.6821 × 10^−1^	4.8619 × 10^−1^
F7	AVG	2.9658 × 10^2^	9.4097 × 10^2^	5.4830 × 10^2^	3.2512 × 10^2^	5.8042 × 10^2^	4.1793 × 10^2^	1.0757 × 10^3^	**2.8929 × 10^2^**
	STD	1.0557 × 10^2^	1.8477 × 10^2^	1.2762 × 10^2^	2.8856 × 10^2^	1.4751 × 10^2^	9.2663 × 10	2.1772 × 10^2^	9.4926 × 10
F8	AVG	5.4627	6.5042	5.9984	5.4877	5.9982	5.6098	6.4208	**5.3501**
	STD	3.6571 × 10^−1^	2.7377 × 10^−1^	2.1249 × 10^−1^	6.8809 × 10^−1^	3.0162 × 10^−1^	4.7670 × 10^−1^	1.6190	2.4341 × 10^−1^
F9	AVG	5.0168	3.3530 × 10^2^	5.5962 × 10^2^	3.4069 × 10	3.3978	3.0946	**2.5553**	4.5696
	STD	1.8876	1.8130 × 10^2^	2.1148 × 10^2^	4.0954 × 10	2.6188 × 10^−1^	3.1284 × 10^−1^	9.5374 × 10^−2^	6.2230
F10	AVG	2.0021 × 10	2.0499 × 10	2.0311 × 10	2.0041 × 10	2.0314 × 10	2.0059 × 10	2.1022 × 10	**1.9994 × 10**
	STD	7.7741 × 10^−1^	1.8716 × 10^−1^	6.6710 × 10^−2^	5.4341 × 10^−2^	1.8459 × 10^−1^	5.6194 × 10^−1^	8.8160 × 10^−2^	7.1465 × 10^−1^

**Table 4:** Wilcoxon rank-sum test of BROMLDE and its competitors on F1–F10 of CEC 2019 benchmark functions.

	BSDE	OMLDE	WDE	PSO	SHADE	JADE	CMAES
F1	+	=	+	+	+	+	+
F2	+	+	+	+	+	−	+
F3	+	+	+	=	+	+	+
F4	+	+	+	+	−	−	+
F5	+	+	+	=	+	=	−
F6	=	+	+	=	+	+	+
F7	=	+	+	=	+	+	+
F8	=	+	+	=	+	+	+
F9	+	+	+	+	=	−	−
F10	=	+	+	+	+	=	+
Statistics Number (+/−/=)	6/0/4	9/0/1	10/0/0	5/0/5	8/1/1	5/3/2	8/2/0

**Table 5:** Results of BROMLDE and other methods for solving 5D functions.

No.	Metric	BSDE	OMLDE	WDE	PSO	SA	J2020	IMODE	BROMLDE
F1	AVG	4.8740 × 10^4^	2.0986 × 10^7^	8.5297 × 10^6^	4.4365 × 10^3^	4.2100 × 10^3^	3.4065 × 10^5^	**8.9505 × 10^2^**	9.0706 × 10^3^
	STD	5.0069 × 10^4^	2.2874 × 10^7^	4.9708 × 10^6^	3.6216 × 10^3^	4.1008 × 10^3^	1.2306 × 10^6^	4.9379 × 10^2^	1.4826 × 10^4^
F2	AVG	1.2414 × 10^3^	1.5199 × 10^3^	1.3496 × 10^3^	1.4760 × 10^3^	1.5509 × 10^3^	1.3121 × 10^3^	**1.2256 × 10^3^**	1.2381 × 10^3^
	STD	5.6217 × 10	9.2984 × 10	9.9400 × 10	1.7947 × 10^2^	1.9126 × 10^2^	1.2762 × 10^2^	5.5404 × 10	6.2432 × 10
F3	AVG	7.0965 × 10^2^	7.2101 × 10^2^	7.2148 × 10^2^	7.1313 × 10^2^	7.3505 × 10^2^	7.1375 × 10^2^	7.0892 × 10^2^	**7.0783 × 10^2^**
	STD	2.0255	5.7317	4.0302	4.8783	1.7421 × 10	4.2279	1.1038	1.1270
F4	AVG	1.9007 × 10^3^	1.9047 × 10^3^	1.9022 × 10^3^	1.9005 × 10^3^	1.9053 × 10^3^	1.9016 × 10^3^	1.9006 × 10^3^	**1.9004 × 10^3^**
	STD	3.3877 × 10^−1^	2.6435	5.6901 × 10^−1^	3.6936 × 10^−1^	3.7258	7.2313 × 10^−1^	1.7476 × 10^−1^	1.1759 × 10^−1^
F5	AVG	1.7167 × 10^3^	3.0457 × 10^3^	1.9032 × 10^3^	5.5299 × 10^3^	2.3202 × 10^3^	3.3174 × 10^3^	**1.7070 × 10^3^**	**1.7070 × 10^3^**
	STD	7.5758	1.0090 × 10^3^	1.2768 × 10^2^	5.2416 × 10^3^	1.1974 × 10^3^	7.0944 × 10^3^	2.8809	1.1347 × 10
F6	AVG	1.6010 × 10^3^	1.6136 × 10^3^	1.6035 × 10^3^	1.6251 × 10^3^	1.7276 × 10^3^	1.6009 × 10^3^	1.6010 × 10^3^	**1.6008 × 10^3^**
	STD	2.6168 × 10^−1^	8.6732	2.0798	3.6268 × 10	9.1687 × 10	6.0805 × 10^−1^	2.2067 × 10^−1^	1.9990 × 10^−1^
F7	AVG	2.1005 × 10^3^	2.1044 × 10^3^	2.1015 × 10^3^	2.1120 × 10^3^	2.1234 × 10^3^	**2.1001 × 10^3^**	**2.1001 × 10^3^**	2.1003 × 10^3^
	STD	1.9307 × 10^−1^	2.9249	5.1746 × 10^−1^	1.5631 × 10	3.1755 × 10	1.9415 × 10^−1^	5.7708 × 10^−2^	1.5899 × 10^−1^
F8	AVG	2.2159 × 10^3^	2.2379 × 10^3^	2.2268 × 10^3^	2.2530 × 10^3^	2.5333 × 10^3^	2.2186 × 10^3^	**2.2046 × 10^3^**	2.2066 × 10^3^
	STD	9.9944	1.0055 × 10	7.1466	4.7534 × 10	3.6440 × 10^2^	1.8780 × 10	3.0763	6.0099
F9	AVG	2.5034 × 10^3^	2.5307 × 10^3^	2.5299 × 10^3^	2.5654 × 10^3^	2.7298 × 10^3^	2.5064 × 10^3^	2.5033 × 10^3^	**2.4993 × 10^3^**
	STD	1.9814 × 10	1.1469 × 10	1.2249 × 10	1.0929 × 10^2^	9.8491 × 10	3.7058 × 10	1.8782	2.3732 × 10
F10	AVG	2.8209 × 10^3^	2.8511 × 10^3^	2.8473 × 10^3^	2.8470 × 10^3^	2.8626 × 10^3^	**2.7933 × 10^3^**	2.8327 × 10^3^	2.8460 × 10^3^
	STD	5.4998 × 10	6.6783	9.4384	1.0499 × 10	7.4130 × 10	7.0970 × 10	5.8544 × 10	4.3048

**Table 6:** Wilcoxon rank-sum test of BROMLDE and its competitors on F1–F10 of CEC 2020 benchmark functions (5D).

	BSDE	OMLDE	WDE	PSO	SA	J2020	IMODE
F1	+	+	+	=	=	=	−
F2	=	+	+	+	+	+	=
F3	+	+	+	+	+	+	+
F4	+	+	+	=	+	+	+
F5	+	+	+	+	+	+	+
F6	+	+	+	+	+	=	+
F7	+	+	+	+	+	−	−
F8	+	+	+	+	+	+	=
F9	=	+	+	=	+	=	=
F10	=	+	+	=	+	−	−
Statistics Number (+/−/=)	7/0/3	10/0/0	10/0/0	6/0/4	9/0/1	5/2/3	4/3/3

**Table 7:** Results of BROMLDE and other methods for solving 10D functions.

No.	Metric	BSDE	OMLDE	WDE	PSO	SA	J2020	IMODE	BROMLDE
F1	AVG	1.4476 × 10^8^	2.4555 × 10^9^	1.5449 × 10^9^	4.0704 × 10^8^	**4.6326 × 10^3^**	3.6412 × 10^7^	1.7090 × 10^7^	4.4096 × 10^7^
	STD	8.3021 × 10^7^	1.3660 × 10^9^	4.9592 × 10^8^	5.1296 × 10^8^	3.7971 × 10^3^	4.3766 × 10^7^	6.3048 × 10^6^	7.2445 × 10^7^
F2	AVG	1.9834 × 10^3^	2.7348 × 10^3^	2.3048 × 10^3^	2.1206 × 10^3^	2.1731 × 10^3^	**1.6655 × 10^3^**	1.9956 × 10^3^	1.8547 × 10^3^
	STD	1.3991 × 10^2^	1.4098 × 10^2^	1.1851 × 10^2^	3.6835 × 10^2^	2.8766 × 10^2^	2.7621 × 10^2^	1.3242 × 10^2^	1.5977 × 10^2^
F3	AVG	7.4407 × 10^2^	7.9016 × 10^2^	8.2616 × 10^2^	7.5025 × 10^2^	7.9052 × 10^2^	7.5559 × 10^2^	7.4235 × 10^2^	**7.3110 × 10^2^**
	STD	5.7745	1.6326 × 10	1.8826 × 10	1.6186 × 10	4.1993 × 10	1.3715 × 10	4.9878	6.5706
F4	AVG	1.9460 × 10^3^	6.6667 × 10^3^	2.1915 × 10^3^	2.0936 × 10^3^	1.9091 × 10^3^	1.9059 × 10^3^	**1.9041 × 10^3^**	1.9299 × 10^3^
	STD	2.6716 × 10	9.4365 × 10^3^	2.5856 × 10^2^	5.5618 × 10^2^	5.2152	1.9810	6.3848 × 10^−1^	4.7804 × 10
F5	AVG	9.7848 × 10^4^	1.5909 × 10^5^	9.7802 × 10^4^	1.9558 × 10^5^	9.7668 × 10^5^	4.4576 × 10^4^	**2.1550 × 10^4^**	5.2912 × 10^4^
	STD	6.6057 × 10^4^	9.6210 × 10^4^	7.5923 × 10^4^	3.3832 × 10^5^	9.0480 × 10^5^	1.3231 × 10^5^	1.5227 × 10^4^	8.1556 × 10^4^
F6	AVG	1.7118 × 10^3^	2.0008 × 10^3^	1.7653 × 10^3^	1.9071 × 10^3^	1.9416 × 10^3^	1.6834 × 10^3^	1.6963 × 10^3^	**1.6636 × 10^3^**
	STD	4.5849 × 10	1.0720 × 10^2^	5.6569 × 10	1.3882 × 10^2^	1.3931 × 10^2^	7.2439 × 10	5.4220 × 10	4.6814 × 10
F7	AVG	8.9530 × 10^3^	2.0206 × 10^4^	1.5814 × 10^4^	9.5733 × 10^3^	2.0077 × 10^6^	1.2221 × 10^4^	4.3612 × 10^3^	**4.3279 × 10^3^**
	STD	5.6729 × 10^3^	2.1511 × 10^4^	8.8941 × 10^3^	8.1418 × 10^3^	2.5879 × 10^6^	3.4438 × 10^4^	1.3724 × 10^3^	1.7359 × 10^3^
F8	AVG	2.3216 × 10^3^	2.5316 × 10^3^	2.4407 × 10^3^	2.3360 × 10^3^	3.1952 × 10^3^	2.3166 × 10^3^	2.3118 × 10^3^	**2.3100 × 10^3^**
	STD	1.9100 × 10	1.0034 × 10^2^	7.0259 × 10	2.0004 × 10	7.9140 × 10^2^	8.2685	5.9606	9.0332
F9	AVG	**2.6409 × 10^3^**	2.7814 × 10^3^	2.7274 × 10^3^	2.7447 × 10^3^	2.8628 × 10^3^	2.7239 × 10^3^	2.7013 × 10^3^	2.6473 × 10^3^
	STD	5.6065 × 10	6.7746 × 10	4.3935 × 10	1.1248 × 10^2^	1.0451 × 10^2^	6.2291 × 10	5.6735 × 10	6.7168 × 10
F10	AVG	2.9502 × 10^3^	3.0807 × 10^3^	3.0229 × 10^3^	2.9518 × 10^3^	3.0048 × 10^3^	2.9424 × 10^3^	**2.9285 × 10^3^**	2.9448 × 10^3^
	STD	8.0811	8.1623 × 10	2.8419 × 10	2.2054 × 10	3.8234 × 10	1.8236 × 10	9.5728	1.0122 × 10

**Table 8:** Wilcoxon rank-sum test of BROMLDE and its competitors on F1–F10 of CEC 2020 benchmark functions (10D).

	BSDE	OMLDE	WDE	PSO	SA	J2020	IMODE
F1	+	+	+	+	−	=	−
F2	+	+	+	+	+	−	+
F3	+	+	+	+	+	+	+
F4	+	+	+	+	=	−	−
F5	+	+	+	=	+	−	=
F6	+	+	+	+	+	=	+
F7	+	+	+	+	+	+	=
F8	+	+	+	+	+	+	+
F9	=	+	+	+	+	+	+
F10	+	+	+	=	+	=	−
Statistics Number (+/−/=)	9/0/1	10/0/0	10/0/0	8/0/2	8/1/1	4/3/3	5/3/2

**Table 9:** Results of BROMLDE and IMODE on F1–F10 of CEC 2020 benchmark functions (20D).

No.	Metric	IMODE		BROMLDE
Unimodal Function/Multimodal Shifted and Rotated Functions				
F1	AVG	**7.7793 × 10^8^**	=	8.0666 × 10^8^
	STD	2.0501 × 10^8^		4.7072 × 10^8^
F2	AVG	**3.5623 × 10^3^**	−	3.9156 × 10^3^
	STD	2.4748 × 10^2^		2.7165 × 10^2^
F3	AVG	8.3820 × 10^2^	+	**8.2117 × 10^2^**
	STD	1.1526 × 10		1.8004 × 10
F4	AVG	**1.9284 × 10^3^**	−	2.1141 × 10^3^
	STD	6.3149		5.7483 × 10^2^
Hybrid Functions				
F5	AVG	7.9308 × 10^5^	+	**6.3443 × 10^5^**
	STD	2.7024 × 10^5^		3.6768 × 10^5^
F6	AVG	2.0119 × 10^3^	=	**1.9989 × 10^3^**
	STD	8.9159 × 10		9.4389 × 10
F7	AVG	4.6835 × 10^5^	+	**2.4124 × 10^5^**
	STD	2.5776 × 10^5^		1.4449 × 10^5^
Composition Functions				
F8	AVG	2.7476 × 10^3^	+	**2.5188 × 10^3^**
	STD	1.5594 × 10^2^		1.2362 × 10^2^
F9	AVG	**2.9273 × 10^3^**	−	2.9362 × 10^3^
	STD	1.2475 × 10		1.4749 × 10
F10	AVG	3.0580 × 10^3^	=	**3.0562 × 10^3^**
	STD	2.4327 × 10		2.6982 × 10
Total Statistics (+/−/=)		4/3/3		

**Table 10:** Influence parameters of the weight of the door.

No.	Variables	Description of Variables
1	$x_{1}$	Thicknesses of B-pillar Inner
2	$x_{2}$	B-pillar Reinforcement
3	$x_{3}$	Floor Side Inner
4	$x_{4}$	Cross Members
5	$x_{5}$	Door Beam
6	$x_{6}$	Door Beltline Reinforcement
7	$x_{7}$	Roof Rail
8	$x_{8}$	Materials of B-pillar Inner
9	$x_{9}$	Floor Side Inner
10	$x_{10}$	Barrier Height
11	$x_{11}$	Hitting Position

**Table 11:** Comparison results of the BROMLDE and its competitors for CSI design problem.

	BSDE	OMLDE	WDE	PSO	SNS	BROMLDE
$x_{1}$	0.5026	0.5943	0.5213	0.5000	0.5400	0.5042
$x_{2}$	1.0778	0.9961	1.0271	1.0302	0.9027	0.9831
$x_{3}$	0.5219	0.5475	0.5115	0.5000	0.5400	0.5178
$x_{4}$	1.2211	1.2961	1.2723	1.2807	1.3599	1.3132
$x_{5}$	0.5648	0.5011	0.5068	0.5724	0.5400	0.5121
$x_{6}$	1.1337	1.3552	1.4657	1.5000	0.5400	1.4363
$x_{7}$	0.5311	0.5754	0.5645	0.5532	0.5400	0.5266
$x_{8}$	0.1920	0.1920	0.1920	0.1920	0.3450	0.1920
$x_{9}$	0.1920	0.1920	0.1920	0.1920	0.3450	0.1920
$x_{10}$	3.3461	−4.9938	−3.5587	1.2682	5.9180	−11.1769
$x_{11}$	7.0345	4.9287	−3.3178	7.0414	20.8016	2.7355
$\Phi_{1}(x)$	−0.3603	−0.4153	−0.4093	−0.3635	−0.3933	−0.4813
$\Phi_{2}(x)$	−3.1044	−3.4401	−2.9162	−2.4132	−0.5110	−2.4075
$\Phi_{3}(x)$	−1.7696	−1.7616	−1.6480	−1.6206	−1.6504	−1.1776
$\Phi_{4}(x)$	−2.3997	−2.9000	−2.5406	−2.0899	−2.4854	−2.8803
$\Phi_{5}(x)$	−0.0710	−0.0771	−0.0733	−0.0661	−0.0771	−0.0737
$\Phi_{6}(x)$	−0.1024	−0.0997	−0.0963	−0.0921	−0.1105	−0.0939
$\Phi_{7}(x)$	−0.0049	−0.0012	−0.0033	−1.6653 × 10^−16^	−0.0030	−4.1458 × 10^−4^
$\Phi_{8}(x)$	−0.0025	−0.0112	−0.0073	−0.0108	−0.0383	−0.0025
$\Phi_{9}(x)$	−0.0274	−0.2094	−0.1592	−0.0187	−0.2601	−0.2999
$\Phi_{10}(x)$	−0.0116	−0.0897	−0.0201	−0.2127	−0.2893	−0.1312
Optimal f(x)	22.6264	23.0179	22.5002	22.4562	22.3046	**22.2372**
AVG	2.3078 × 10	2.4141 × 10	2.3283 × 10	2.4435 × 10	2.3069 × 10	**2.2463** **× 10**
STD	2.3420 × 10^−1^	4.8138 × 10^−1^	3.9897 × 10^−1^	1.0472	4.4591 × 10^−1^	**1.4463** **× 10^−1^**
+/−/=	+	+	+	+	+	

**Table 12:** Influence variables of the SR problem.

No.	Variables	Descriptions
1	$b(=x_{1})$	Face Width
2	$m(=x_{2})$	Module of Teeth
3	$z(=x_{3})$	The Number of Teeth in the Pinion
4	$l_{1}(=x_{4})$	Length of the First Shaft Between Bearings
5	$l_{2}(=x_{5})$	Length of the Second Shaft Between Bearings
6	$d_{1}(=x_{6})$	The Diameter of First Shafts
7	$d_{2}(=x_{7})$	The Diameter of Second Shafts

**Table 13:** Comparison results of the BROMLDE and its competitors for SR design problem.

	BSDE	OMLDE	WDE	PSO	SNS	BROMLDE
$x_{1}$	3.5031	3.5183	3.5027	3.5050	3.5122	3.5022
$x_{2}$	0.7003	0.7000	0.7000	0.7000	0.7010	0.7001
$x_{3}$	28	28	28	28	28	28
$x_{4}$	7.4203	8.0487	7.3417	7.8632	7.3010	7.3106
$x_{5}$	7.7615	7.7408	7.7770	7.7717	7.7685	7.7838
$x_{6}$	3.3619	3.3534	3.3479	3.3501	3.3599	3.3464
$x_{7}$	5.2912	5.2889	5.3032	5.2858	5.2878	5.2864
$\Phi_{1}(x)$	−21.1076	−21.2716	−21.0613	−21.0881	−21.3246	−21.0638
$\Phi_{2}(x)$	−949.5132	−954.1054	−948.2157	−948.9666	−955.5880	−948.2858
$\Phi_{3}(x)$	−4.2014	−2.8237	−4.2928	−3.1479	−4.4976	−4.3613
$\Phi_{4}(x)$	−30.9423	−31.1344	−31.0309	−30.6659	−30.7999	−30.5336
$\Phi_{5}(x)$	−15.2784	−6.5270	−1.6458	−3.4119	−13.4128	−0.1271
$\Phi_{6}(x)$	−2.5637	−1.4703	−8.3399	−3.2401 × 10^−11^	−0.9238	−0.2888
$\Phi_{7}(x)$	−20.3907	−20.4000	−20.3991	−20.4000	−20.3720	−20.3972
$\Phi_{8}(x)$	−0.0020	−0.0262	−0.0036	−0.0071	−0.0102	−0.0024
$\Phi_{9}(x)$	−6.9980	−6.9738	−6.9964	−6.9929	−6.9898	−6.9976
$\Phi_{10}(x)$	−0.4774	−1.1185	−0.4198	−0.9381	−0.3611	−0.3911
$\Phi_{11}(x)$	−0.0412	−0.0230	−0.0435	−0.0573	−0.0520	−0.0687
Optimal f(x)	5.4532 × 10^3^	5.4675 × 10^3^	5.4531 × 10^3^	5.4491 × 10^3^	5.4672 × 10^3^	**5.4421** **× 10^3^**
AVG	5.4666 × 10^3^	5.5240 × 10^3^	5.4708 × 10^3^	5.5495 × 10^3^	5.4829 × 10^3^	**5.4660** **× 10^3^**
STD	8.1468	3.4116 × 10	9.5649	8.1021 × 10	5.6624	1.7858 × 10
+/−/=	=	+	=	+	+	
